# Isogenic induced pluripotent stem cell line ICGi036-A-1 from a patient with familial hypercholesterolaemia, derived by correcting a pathogenic variant of the gene LDLR c.530C>T

**DOI:** 10.18699/vjgb-25-22

**Published:** 2025-04

**Authors:** A.S. Zueva, A.I. Shevchenko, S.P. Medvedev, E.A. Elisaphenko, A.A. Sleptcov, M.S. Nazarenko, N.A. Tmoyan, S.M. Zakian, I.S. Zakharova

**Affiliations:** Institute of Cytology and Genetics of the Siberian Branch of the Russian Academy of Sciences, Novosibirsk, Russia Novosibirsk State University, Novosibirsk, Russia; Institute of Cytology and Genetics of the Siberian Branch of the Russian Academy of Sciences, Novosibirsk, Russia; Institute of Cytology and Genetics of the Siberian Branch of the Russian Academy of Sciences, Novosibirsk, Russia; Institute of Cytology and Genetics of the Siberian Branch of the Russian Academy of Sciences, Novosibirsk, Russia; Institute of Cytology and Genetics of the Siberian Branch of the Russian Academy of Sciences, Novosibirsk, Russia Research Institute of Medical Genetics, Tomsk National Research Medical Center of the Russian Academy of Sciences, Tomsk, Russia; Institute of Cytology and Genetics of the Siberian Branch of the Russian Academy of Sciences, Novosibirsk, Russia Research Institute of Medical Genetics, Tomsk National Research Medical Center of the Russian Academy of Sciences, Tomsk, Russia; Institute of Cytology and Genetics of the Siberian Branch of the Russian Academy of Sciences, Novosibirsk, Russia National Medical Research Center of Cardiology named after academician E.I. Chazov, Moscow, Russia; Institute of Cytology and Genetics of the Siberian Branch of the Russian Academy of Sciences, Novosibirsk, Russia; Institute of Cytology and Genetics of the Siberian Branch of the Russian Academy of Sciences, Novosibirsk, Russia

**Keywords:** familial hypercholesterolaemia, LDLR, induced pluripotent stem cells, genome editing, sogenic cell lines, семейная гиперхолестеринемия, LDLR, индуцированные плюрипотентные стволовые клетки, геномное редактирование, изогенные линии клеток

## Abstract

Familial hypercholesterolaemia is a common monogenic disorder characterized by high plasma cholesterol levels leading to chronic cardiovascular disease with high risk and often early manifestation due to atherosclerotic lesions of the blood vessels. The atherosclerotic lesions in familial hypercholesterolaemia are mainly caused by pathogenic variants of the low-density lipoprotein receptor (LDLR) gene, which plays an important role in cholesterol metabolism. Normally, cholesterol-laden low-density lipoproteins bind to the LDLR receptor on the surface of liver cells to be removed from the bloodstream by internalisation with hepatocytes. In familial hypercholesterolaemia, the function of the receptor is impaired and the uptake of low-density lipoproteins is significantly reduced. As a result, cholesterol accumulates in the subendothelial space on the inner wall of blood vessels, triggering atherogenesis, the formation of atherosclerotic plaques. At present, there are no effective and universal approaches to the diagnosis and treatment of familial hypercholesterolaemia. A relevant approach to study the molecular genetic mechanisms of the disease and to obtain systems for screening chemical compounds as potential drugs is the generation of cellular models based on patient-specific induced pluripotent stem cells. The aim of our work was to derive an isogenic genetically modified induced pluripotent stem cell line by correcting the pathogenic allelic variant c.530C of the LDLR gene in the original iPSC previously obtained from a compound heterozygote patient with familial hypercholesterolaemia. The resulting isogenic iPSC line differs from the original by only one corrected nucleotide substitution, allowing us to study the direct effect of this pathogenic genetic variant on physiological changes in relevant differentiated cells. CRISPR/Cas-mediated base editing was used to correct the single nucleotide substitution. The resulting genetically modified iPSC line has pluripotency traits, a normal karyotype, a set of short tandem repeats identical to that in the original line and can be used to obtain differentiated derivatives necessary for the elaboration of relevant cell models.

## Abstract (ru)

Семейная гиперхолестеринемия является распространенным моногенным заболеванием, которое характеризуется повышенным содержанием холестерина в плазме крови, приводящим к хроническим заболеваниям сердечно-сосудистой системы с высоким риском и ранним проявлением развития патологий, вызван- ных атеросклеротическими поражениями кровеносных сосудов. Образование атеросклеротических бляшек при семейной гиперхолестеринемии в основном обусловлено патогенными вариантами гена рецептора липопротеинов низкой плотности LDLR (low-density lipoprotein receptor), играющего важную роль в метаболизме холестерина. В норме липопротеины низкой плотности, переносящие холестерин, связываются с рецептором LDLR на поверхности клеток печени и выводятся из кровотока путем интернализации гепатоцитами. При семейной гиперхолестеринемии происходит нарушение функционирования рецептора и значительное сниже- ние интернализации липопротеинов низкой плотности. Это приводит к их накоплению в субэндотелиальном пространстве внутренней стенки кровеносных сосудов и вызывает атерогенез – образование атеросклеротических бляшек. На сегодняшний день не существует эффективных и универсальных подходов к диагностике и лечению семейной гиперхолестеринемии. Актуальным подходом для исследования молекулярно-генетических особенностей заболевания и разработки систем скрининга химических соединений – потенциальных лекарственных препаратов – является создание клеточных моделей на основе индуцированных плюрипотентных стволовых клеток (ИПСК) пациентов. Целью нашей работы было создание изогенной генетически моди- фицированной линии индуцированных плюрипотентных стволовых клеток путем коррекции патогенного аллельного варианта c.530C гена LDLR в линии ИПСК, полученной ранее от пациента-компаундной гетерозиготы с семейной гиперхолестеринемией. Созданная изогенная клеточная линия ИПСК отличается от исходной только одной скорректированной нуклеотидной заменой, что позволяет исследовать непосредственное влияние данного патогенного генетического варианта на физиологические изменения в релевантных дифференцированных клетках. Для коррекции однонуклеотидных замен использован CRISPR/Cas9-опосредованный метод редактирования оснований. Полученная генетически модифицированная линия ИПСК обладает свойствами плюрипотентности, имеет нормальный кариотип, идентичный исходной линии набор коротких тандемных повторов и может быть использована для формирования дифференцированных производных, необходимых при разработке релевантных клеточных моделей.

## Introduction

Despite advances in medical technology and revolutionary discoveries in biology, cardiovascular disease, caused mainly by atherosclerotic lesions, remains the leading cause of death worldwide, according to the World Health Organisation.

The most common inherited disease leading to atherosclerosis is familial hypercholesterolaemia (FH) (Zakharova et al., 2024b). According to the European Atherosclerosis Society, this disease occurs with a high frequency, namely one in 250 people for the heterozygous form, one in 300 thousand to one in 1 million people for the homozygous form (Ezhov et al., 2019). Nevertheless, the disease can have a latent course with difficult diagnosis and manifest acute vascular catastrophes in the form of heart attacks, strokes and other ischaemic lesions, often leading to death (Hopkins et al., 2011; Talmud et al., 2014; Ference et al., 2017). Currently, up to 70 % of suspected heterozygous carriers remain undiagnosed1 (Rau et al., 2023).


Supplementary Materials are available in the online version of the paper:https:// https:// familyheart.org/familial-hypercholesterolemia



Information portal of the biotech genetic testing company “23andMe https://www.23andme.com/topics/health-predispositions/fh/?srsltid=AfmBOooDFqM2USz3G0j9PZg-ng-15q__ dvPbcQL6qgCzJOQodQhsLil7


FH is an autosomal dominant disorder associated with elevated low-density lipoprotein (LDL) cholesterol levels and a high risk of premature cardiovascular disease (CVD) (Harada-Shiba, 2023). FH can be heterozygous or homozygous. Patients with the homozygous FН form usually show early CVD manifestations, and without serious comprehensive therapy, their life expectancy does not exceed 30 years (Hopkins et al., 2011). FH is caused by pathogenic allelic variants in genes encoding key proteins involved in LDL clearance mediated by the LDLR (low-density lipoprotein receptor) (Gu et al., 2024). In 85 % of diagnosed FH patients, pathological conditions are caused by a disruption of the LDLR gene, which encodes the low-density lipoprotein receptor on the surface of hepatocytes (Hendricks-Sturrup et al., 2020). FH is rarely associated with de novo pathogenic allelic variants (Fularski et al., 2024). In this context, cascade genetic screening of relatives and prevention of atherosclerosis in diagnosed carriers is important.

Despite the high prevalence of FH, there are no effective treatments. According to data published by the European Atherosclerosis Society in 2022, less than 3 % of patients worldwide achieve cholesterol-lowering goals with medications (Ray et al., 2022; Harada-Shiba, 2023). The lack of effective treatment for FH is linked to the lack of relevant models for both drug trials and the study of FH pathogenesis.

A promising approach to investigate the molecular genetic basis of the disease is the generation of isogenic iPSC lines from patients with FH. Previously, we obtained the ICGi036-A iPSC line from a compound heterozygous patient with familial hypercholesterolemia. This line is registered in the Human Pluripotent Stem Cell Registry (hPSCreg) with the identifier RRID:CVCL_B5EJ (Zakharova et al., 2022a). The initial iPSC line ICGi036-A contains two allelic variants of the LDLR gene, which are missense mutations c.530C>T (p.Ser177Leu) and c.1054T>C (p.Cys352Arg).

The c.530C>T substitution, rs121908026 (p.Ser177Leu), is located in exon 4 of the LDLR gene (Semenova et al., 2020; Meshkov et al., 2021). This missense mutation results in the substitution of serine for leucine at codon 177 in the highly conserved SerAspGlu sequence in the ligand-binding domain of LDLR (Südhof et al., 1985). This substitution slows the transport of LDLR protein to the cell surface, resulting in defective receptors being unable to bind cholesterolladen LDLRs, which leads to a reduction in LDL capture to approximately 6–31 % (Thormaehlen et al., 2015). The LDLR(NM_000527.5):c.530C>T allelic variant is reported in databases as a pathogenic variant causing FH (ClinVar ID 3686; OMIM:606945.0004; UniProt variants VAR_005327; VarSome http://varso.me/1dmA).

The c.1054T>C substitution, rs879254769 (p.Cys352Arg), is located in exon 7 of the LDLR gene and encodes cysteine instead of arginine at codon 352 in an epidermal growth factor-like domain (Semenova et al., 2020; Meshkov et al., 2021). The allelic variant LDLR(NM_000527.5):c.1054T>A is reported in databases as pathogenic/likely pathogenic, causing FH (ClinVar ID 251618; VarSome http://varso.me/0J8J).

There is evidence that the c.530C>T and c.1054T>C LDLR allelic variants can independently cause FH. For example, the heterozygous c.530C>T substitution in LDLR is associated with FH in several countries including the Czech Republic, India, Portugal, Poland and Spain (Bourbon et al., 2008; Palacios et al., 2012; Tichý et al., 2012; Setia et al., 2016; Sharifi et al., 2016). In addition, this allelic variant in compound heterozygosity with EX7_EX10del (c.941-?_1186+?del) in the LDLR gene has been reported in Brazil, and together with c.55G>C (p.Asp19His) within the ABCG8 gene in FH patients in Malaysia (Jannes et al., 2015; Mohd Nor et al., 2019).

The c.1054T>C LDLR allelic variant has been found in heterozygous form in FH patients in Taiwan and Russia, and as a compound heterozygote together with c.796G>A (p.Asp266Asn) in an FH patient in Western Siberia (Meshkov et al., 2021; Shakhtshneider et al., 2021; Huang et al., 2022). In addition, our previous study confirmed positioning in trans between the c.530C>T and c.1054T>C allelic variants in FH patient-specific ICGi036-A iPSCs (Nazarenko et al., 2023).

In the present work, we derive and perform a detailed characterisation of the isogenic genetically modified iPSC line ICGi036-A-1 by base editing correction of LDLR allelic variants in the original iPSC line previously obtained from a compound heterozygous FH patient carrying pathogenic c.530C>T (p.Ser177Leu) and likely pathogenic c.1054T>C (p.Cys352Arg) LDLR alleles. The genetically modified iPSC line with the corrected allelic variant c.530C>T (p.Ser177Leu) can be used to obtain relevant cell types for FH modelling and drug development.

## Materials and methods

Cell lines. The human PSC lines used in the present work are listed below:

• iPSC line ICGi036-A (RRID: CVCL_B5EJ) from a compound heterozygous FH patient with two LDLR allelic variants, namely pathogenic c.530C>T (p.Ser177Leu), rs121908026, ClinVar ID 3686, OMIM:606945.0004 and likely pathogenic c.1054T>C (p.Cys352Arg), rs879254769, ClinVar ID 251618. The previously obtained iPSC line (Zakharova et al., 2022a) was used to derive an isogenic genetically modified iPSC; • healthy donor iPSC line ICGi022-A (RRID: CVCL_ZE02) (Malakhova et al., 2020), for pluripotency marker control; • healthy donor embryonic stem cells (ESCs) HuES9 (HVRDe009-A) (RRID: CVCL_0057) (Cowan et al., 2004), for pluripotency marker control.

IPSCs and ESCs cultivation. IPSCs and ESCs were incubated in DMEM/F12 growth medium containing 15 % KnockOut SR (Thermo Fisher Scientific), 1 mM GlutaMax (Thermo Fisher Scientific), 1 % NEAA (Thermo Fisher Scientific), 0.25 mM 2-mercaptoethanol (Thermo Fisher Scientific) and 10 ng/ml bFGF (Sci-store). Cells were grown on a layer of mitotically inactivated mouse embryonic fibroblasts. IPSCs were cultured in an incubator at 37 °C and 5 % CO2. IPSC and ESC colonies were enzymatically dissociated using TrypLE (Thermo Fisher Scientific) and plated in fresh medium with the addition of 2 μM ROCK inhibitor thiazovivin (STEMCELL Technologies) every 5 days.

**Fig. 1.**
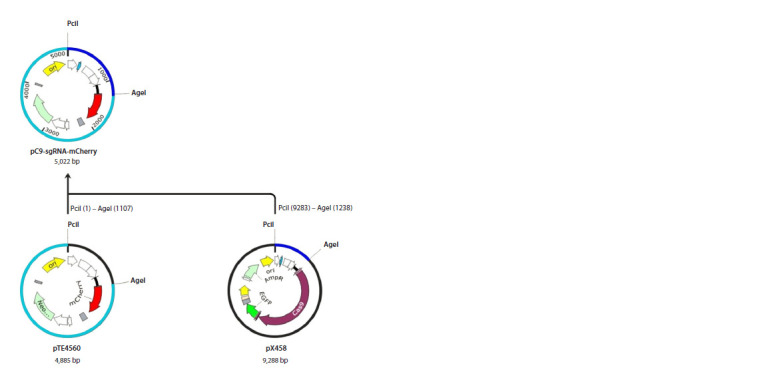
Design of pC9-sgRNA-mCherry plasmid assembly from plasmids pTE4560 and pX458 in SnapGene software.

Vectors for genetic correction of iPSCs. To insert the spacer sequence for the guide RNA, we created a universal plasmid pC9-sgRNA-mCherry (Fig. 1), allowing its delivery into cells to be detected by the mCherry fluorescence signal.

Target DNA fragments were obtained by digestion with PciI and AciGI endonucleases (SibEnzyme) and combined using phage T4 DNA ligase. One 1,244 bp fragment, isolated from plasmid pX458 (addgene #48138), contains the U6 promoter sequence and a site for spacer cloning. Another 3,778 bp fragment, derived from plasmid pTE4560 (addgene #107526), includes the kanamycin antibiotic resistance gene and the mCherry fluorescent protein sequence. The final assembly of plasmid pC9-sgRNA-mCherry was confirmed by restriction analysis and Sanger sequencing at the Genomics Collective Use Centre of the Siberian Branch of the Russian Academy of Sciences (http://www.niboch.nsc.ru/doku.php/sequest).

Oligonucleotides for guide RNAs were selected using PnB Designer (https://fgcz-shiny.uzh.ch/PnBDesigner/) (Siegner et al., 2021).

Guide RNA expression from the U6 promoter is enhanced when nucleotide G is located immediately after the 5′- CACC- 3′ sequence and before the spacer sequence (Bauer et al., 2015). Based on this, we added nucleotide G to the 5′ end of the selected oligonucleotides and then generated a complementary oligonucleotide sequence. Next, the sequence 5′-CACC-3′ was added to the 5′ end of the upper DNA strand and 5′-AAAC-3′ was added to the 5′ end of the complementary oligonucleotide sequence for subsequent integration into the pC9-sgRNA-mCherry vector through BpiI endonuclease restriction sites (see the Table).

**Table 1.**
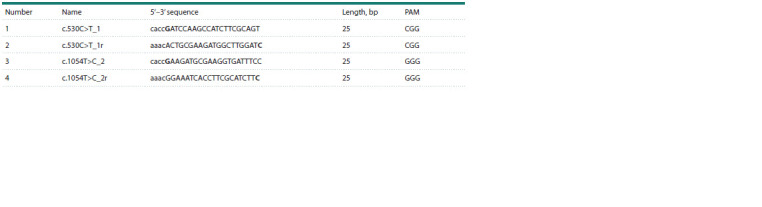
Oligonucleotide sequences for guide RNAs modified for insertion into the plasmid vector Notе. Capital letters indicate oligonucleotides for guide RNAs selected using PnB Designer (https://fgcz-shiny.uzh.ch/PnBDesigner/) (Siegner et al., 2021), bold letters indicate nucleotides added to increase the expression level from the U6 promoter, lowercase letters indicate added sticky ends, and “r” indicates generated complementary oligonucleotide sequences).

Oligonucleotides were synthesised by Biosset (https:// www.biosset.com/). Single-stranded oligonucleotides were phosphorylated at the 5′ end using phage T4 polynucleotide kinase (New England Biolabs) and annealed to form doublestranded molecules. Using phage T4 ligase, the resulting double-stranded molecules with sticky ends were inserted into the guide RNA spacer sites of the BpiI endonuclease prelinearised pC9-sgRNA-mCherrys plasmid. Sanger sequencing was used to confirm that the resulting plasmids contained integrated target sequences

Two two-component plasmid systems were used for correction of targeted single-nucleotide substitutions. System 1 for correction of the c.530C>T substitution consists of the plasmid vector xCas9(3.7)-ABE(7.10) (addgene #108382), encoding adenine deaminase and xCas9n nickase (adenine base editor), and the plasmid pC9-sgRNA-mCherry with an integrated guide RNA spacer sequence. System 2 for correction of the c.1054T>C substitution consists of the plasmid vector xCas9(3.7)-BE4 (addgene #108381) encoding a cytidine deaminase, xCas9n nickase (cytidine base editor), and also the plasmid pC9-sgRNA-mCherry with an integrated guide RNA spacer sequence

Plasmid DNA isolation for subsequent lipofection was performed using the HiPure Plasmid EF Midi Kit (Magen).

Delivery of vectors to correct the c.530C>T and c.1054T>C substitutions of the LDLR gene sequence. Delivery of plasmids encoding base editors and guide RNAs into iPSCs was performed by lipofection using the Lipofectamine 3000 Transfection Reagent Kit (Thermo Fisher Scientific) according to the manufacturer’s protocol with modifications. Twenty-four hours before lipofection, iPSCs cultured on mitotically inactivated mouse embryonic fibroblasts were plated into three wells of a 12-well plate coated with Matrigel matrix (Corning) in medium containing 15 % koSR (Thermo Fisher Scientific) and 2 μM ROCK inhibitor thiazovivine (STEMCELL Technologies). To increase the efficiency of upcoming lipofection, iPSCs were disaggregated to a single cell state during passaging using TrypLE. 4–5 hours after re-plating, when the cells had attached to the culture surface, the medium was changed to a serum-free medium containing thiazovivine and a 3-fold increased bFGF amount (30 ng/ml). 2 hours before lipofection, the medium was changed to an equivalent fresh medium without thiazovivine.

Lipofection was performed in medium without koSR and thiazovivine and with 30 ng/ml bFGF. The ratio between the amount of base-editor plasmids and plasmids with guide RNA was 3:1 in ng. Prior to lipofection, 150 μL Opti-MEM medium (Thermo Fisher Scientific) and 9 μL Lipofectamine 3000 were mixed in tube 1. In tube 2, 150 μl Opti-MEM medium, 6 μl P 3000 reagent, 125 ng each of plasmid DNA pC9-1054_2-mCherry and pC9-530-mCherry, 375 ng each of plasmid DNA xCas9(3.7)-ABE(7.10) and xCas9(3.7)-BE4 were added. The contents of tubes 1 and 2 were mixed and incubated for 15 minutes at room temperature. We added 100 μl of the mixture to 1 × 105 iPSCs growing in one well of a 12-well plate in 1 ml of medium. 26 hours after lipofection, the growth medium was removed and fresh medium containing 15 % koSR, 30 ng/ml bFGF and thiazovivin was added. The red signal of mCherry protein was detected between 24 and 48 hours after lipofection using a Nikon TiE inverted fluorescence microscope

Selection and subcloning of the resulting iPSC clones. 48 hours after delivery of the base editing system to iPSCs, cells were disaggregated and selected by flow cytometry using the red signal of the fluorescent protein mCherry on a Sony MA900 instrument. The resulting single cell suspension of iPSCs was seeded onto culture surfaces with mitotically inactivated mouse embryonic fibroblasts. For subsequent subcloning and analysis, individual iPSC colonies grown from isolated selected cells were mechanically harvested using glass capillaries and transferred to individual tissue culture wells pre-seeded with mitotically inactivated mouse embryonic fibroblasts.

Analysis of LDLR gene editing results in selected iPSC clones. The editing results of the c.530C>T and c.1054T>C substitutions in the LDLR sequence in selected iPSC clones were analysed by PCR with subsequent Sanger sequencing. Genomic DNA was isolated from iPSCs using QuickExtract DNA Extraction Solution reagent (Lucigen) according to the manufacturer’s instructions. PCR was performed using the BioMaster HS-Taq PCR-Color (2×) kit (Biolabmix) on a T100 thermal cycler amplifier (Bio-Rad). Programme parameters were as follows: 98 °C – 30 seconds; 98 °C – 15 seconds, 60 °C – 15 seconds, 72 °C – 30 seconds, 35 cycles; 72 °C – 5 minutes. Primer sequences are given in Table S1 in Supplementary Material2.


Tables S1–S4 are available at: https://vavilov.elpub.ru/jour/manager/files/Suppl_Zueva_Engl_29_2.pdf


Oligonucleotides were synthesised in Biosset (https://www. biosset.com/). Sanger sequencing reactions were performed using Big Dye Terminator V.3.1 Cycle Sequencing Kit (Applied Biosystems) and analysed at the Genomics Collective Use Centre of the Siberian Branch of the Russian Academy of Sciences (http://www.niboch.nsc.ru/doku.php/sequest) on an ABI3130XL genetic analyser.

Mycoplasma and episome detection. Testing for mycoplasma and episome contamination was performed by PCR as previously described (Choppa et al., 1998; Okita et al., 2013). Primer sequences are provided in Table S1. Program parameters for episome detection were as follows: 95 °C – 5 minutes; 95 °C – 15 seconds, 58 °C – 15 seconds, 72 °C – 20 seconds, 35 cycles; 72 °C – 5 minutes; for mycoplasma detection: 95 °C – 3 minutes; 95 °C – 15 seconds, 67 °C – 15 seconds, 72 °C – 20 seconds, 35 cycles; 72 °C – 5 minutes.

Karyotyping. Genetically modified iPSCs were karyotyped at passage 15 according to the previously described protocol using DAPI banding according to the International System of Human Cytogenetic Nomenclature (Grigor’eva et al., 2024).

STR analysis. Short tandem repeat (STR) analysis was performed with Genoanalytica (https://www.genoanalytica. ru). iPSC DNA samples were genotyped by PCR using COrDIS EXPERT 26 direct amplification reagent kit (Russia) according to the manufacturer’s protocol with subsequent separation of amplicons on a 3130 Genetic Analyzer capillary electrophoresis instrument (HITACHI, Applied Biosystems Group of The Applera Corporation, Japan, USA, Registration Certificate No. FSZ 2004/1586). Electropherograms with amplicon patterns are available on request from the authors.

Quantitative RT-PCR. To analyse pluripotency gene expression, RNA was isolated from cells using Trizol reagent (Thermo Fisher Scientific) according to the manufacturer’s protocol. DNAase I treatment was performed using DNA-free kit (Thermo Fisher Scientific). RNA reverse transcription was performed using M-MuLV reverse transcriptase kit (Biolabmix) and random hexamer primers (Thermo Fisher Scientific) according to the manufacturer’s protocol.

Real-time PCR was used to analyse the relative expression levels of pluripotency genes (OCT4, NANOG, SOX2) in genetically modified and initial isogenic iPSC lines with normalisation to two housekeeping genes: ACTB and B2M. Primer sequences are given in Table S1. Reactions were performed using the BioMaster HS-qPCR SYBR Blue (2×) kit (Biolabmix) on a T100 thermal cycler amplifier (Bio-Rad). Program parameters were as follows: 98 °C – 30 seconds; 98 °C – 15 seconds, 60 °C – 15 seconds, 72 °C – 30 seconds, 35 cycles; 72 °C – 5 minutes. For each sample, three biological and two technical replicates were analysed.

The results were evaluated with qBase+ software (CellCarta https://cellcarta.com/genomic-data-analysis/) using the generalised ΔΔCt method, taking into account the reaction efficiency calculated from the results of a six-point calibration curve.

Immunofluorescence staining. Cell preparation and antibody precipitation were performed according to the previously described protocol (Vaskova et al., 2015). Briefly, cells were fixed in 4 % formaldehyde for 10 minutes, permeabilised with 0.5 % Triton X-100 for 30 minutes (this step was omitted for surface antigens) and blocked with 1 % bovine serum albumin solution in 1X PBS. All procedures were carried out at room temperature. Incubation with primary antibodies was performed overnight at 4 °C. Secondary antibodies were incubated with cells for 1 hour in the dark at room temperature. Cell nuclei were counterstained with DAPI. Visualisation and imaging of samples were performed on a Ti-E inverted fluorescence microscope (Nikon) using NIS Advanced Research software. The list of primary and secondary antibodies is given in Table S2.

Spontaneous differentiation in vitro. To confirm pluripotency of genetically modified iPSCs, they were induced to differentiate into embryoid bodies within 14 days. The resulting embryoid bodies were seeded onto Matrigel (Corning)-coated Chambered Coverglass plates (Thermo Fisher Scientific) and cultured until the 21st day.

## Results

In this study, we obtained a genetically modified iPSC line ICGi036-A-1 with a corrected pathogenic allelic variant c.530C>T of the LDLR gene, originally derived from a compound heterozygous patient associated with FH. To correct this pathogenic variant, we used the base editing technique, for which we constructed an episomal vector pC9-sgRNAmCherry containing a universal site for cloning any guide RNA spacers of the CRISPR/Cas systems and the gene encoding mCherry red fluorescent protein. The constructed plasmid allows visualisation of transfection results within 24–48 hours and selection of cells by flow cytometry upon delivery of the editing system into the cell

**Fig. 2.**
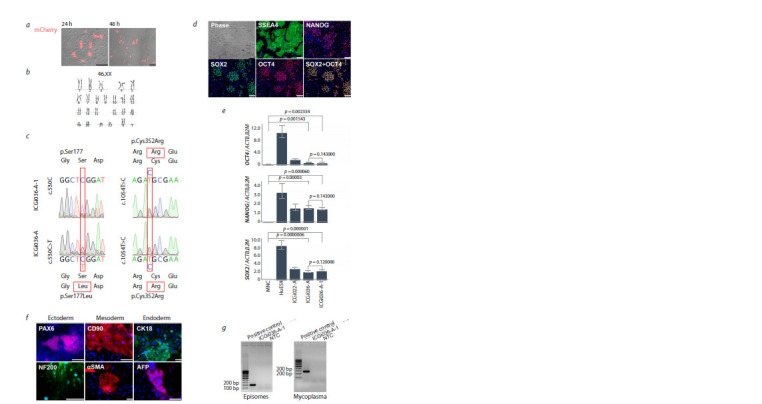
Characteristics of the isogenic genetically modified iPSC line ICGi036-A-1 with the corrected pathogenic allelic variant c.530C>T. a – mCherry protein fluorescence in iPSCs 24 and 48 h after transfection; b – karyotype in the resulting iPSC line; c – chromatograms demonstrating the corrected c.530C position in the ICGi036-A-1 line; d – colony morphology and staining with antibodies against pluripotency markers OCT4, NANOG, SOX2 and SSEA4 in the ICGi036-A-1 line; e – quantification of pluripotency gene expression of OCT4, NANOG and SOX2 by real-time PCR; f – ICGi036-A-1 line retains the ability to differentiate into three germ layer derivatives; g – absence of episomal vectors and mycoplasma contamination in the ICGi036-A-1 line. NTC – no template control. The scale bar for all images is 100 μm.

Guide RNA spacer sequences were inserted into the universal vector containing mCherry. This resulted in two plasmids: pC9-530-mCherry to correct the c.530C>T substitution and pC9-1054_2-mCherry to correct the c.1054T>C substitution in the LDLR gene. The resulting guide RNA plasmids, together with plasmids encoding adenine and cytidine base editor sequences, were lipofected into the ICGi036-A iPSC line (Zakharova et al., 2022a). After 24 and 48 hours, cells with red mCherry protein fluorescence were detected, indicating successful delivery and function of the plasmids (Fig. 2a). We selected cells with mCherry fluorescence by flow cytometry and obtained 96 clones.

We examined selected clones by Sanger sequencing and identified the subclone 130S5, in which the c.530C>T position of the pathogenic allelic variant of the LDLR gene was corrected to the c.530C position corresponding to the reference sequence of the gene (Fig. 2c). The c.1054T>C substitution was not corrected.

Short tandem repeat (STR) analysis showed that the resulting iPSC line ICGi036-A-1 matched the original isogenic line ICGi036-A and patient mononuclear cells at 26 polymorphic loci (Zakharova et al., 2022a) (Table S3).

To confirm that the resulting genetically modified ICGi036- A-1 iPSCs retained self-renewal and pluripotency properties, we examined their pluripotency markers and ability to form three germ layer derivatives. Immunofluorescence staining with antibodies against the transcription factors OCT4, NANOG, SOX2 and the surface antigen SSEA4 showed that all colonies of 130S5 iPSCs were positive for these markers (Fig. 2d). Analysis of the relative expression of the pluripotency genes OCT4, NANOG, SOX2 by real-time PCR showed that their expression levels did not differ significantly from the isogenic control, the original line ICGi036-A (Fig. 2e). At the same time, the genetically modified ICGi036-A-1 iPSC line displayed a significantly higher expression level of pluripotency genes compared to mononuclear cells (MNCs) from which the original isogenic ICGi036-A iPSC line was derived. Analysis of spontaneous differentiation of the ICGi036-A-1 line revealed a heterogeneous population of cells, among which immunofluorescence staining revealed derivatives positive for markers attributable to ectoderm (PAX6, NF200), mesoderm (CD90, αSMA) and entoderm (CK18, AFP) (Fig. 2f ). Thus, the genetically modified iPSC line demonstrates the ability to give rise to derivatives of three primary germ layers, which is a key property of pluripotent stem cells

Genetically modified ICGi036-A-1 iPSCs have a normal diploid karyotype: 46,XX (Fig. 2b). Analysis of 23 polymorphic short tandem repeat loci validated the identity of ICGi036- A-1 iPSCs and the original isogenic ICGi036-A iPSCs. The obtained iPSCs lacked episomal vectors at passage 10 and mycoplasma contamination at passage 25 (Fig. 2g).

The passport of the cell line obtained is shown in Table S4.

## Discussion

Advances in the technologies associated with the generation and application of induced pluripotent stem cells (iPSCs) have opened up new avenues for biological research and biomedical innovations. iPSCs are being used for human disease modelling, high-throughput drug screening and the development of advanced biomedical cell therapy products due to their available minimally invasive derivation method, unlimited proliferative potential and the ability to direct differentiation into all adult cell types (Cerneckis et al., 2024).

Patient-specific cell models derived from differentiated iPSCs help to understand the molecular genetic basis of disease and to develop more effective targeted therapies (Brooks et al., 2022). For example, our group has previously obtained iPSCs from patients with familial hypercholesterolaemia carrying pathogenic allelic variants in the LDLR gene (Zakharova et al., 2022a–c). Using directed iPSC differentiation, we first derived endothelial cells with LDLR pathology, modelling FH (Zakharova et al., 2024a). We found that endotheliocytes derived from FH patient-specific iPSCs, although not exposed to oxidative stress, have impaired LDLR receptor function and show signs of endothelial dysfunction. The data obtained contribute to the understanding of the moleculargenetic mechanisms underlying FH-related atherosclerosis

The combination of iPSC technologies and genome editing methods provides isogenic cell models with similar genetic backgrounds, allowing physiological changes to be reliably studied in relevant differentiated cells (Niemitz, 2014; Omer et al., 2017; Kawatani et al., 2021; Liang et al., 2022; Wang et al., 2022; Chai et al., 2023; Bonnycastle et al., 2024). Isogenic iPSC systems can be generated either by altering the DNA sequence of healthy donor cells or by correcting a pathogenic allelic variant in patient-specific iPSCs (Nandy et al., 2023; Pavlova et al., 2023). In this work, we used CRISPR/Cas9- mediated base editing to generate a genetically modified line ICGi036-A-1, an isogenic line of ICGi036-A iPSCs from a patient heterozygous for the pathogenic and likely pathogenic allelic variants of the LDLR gene with FH (Zakharova et al., 2022a). In the resulting genetically modified line ICGi036- A-1, the pathogenic allelic variant c.530C>T was corrected to the reference c.530C. The iPSC line retains pluripotency, has a normal karyotype and is identical to the original isogenic iPSC line ICGi036-A by the set of short tandem repeats.

In the resulting isogenic iPSC line ICGi036-A-1, the second position, c.1054T>C, remained uncorrected. The status of this allelic variant is currently defined as “pathogenic/likely pathogenic”. The study of differentiated derivatives from heterozygous iPSCs with a corrected c.530C position will help to clarify the status of the c.1054T>C position.

We used the base editing method to correct single nucleotide substitutions in the LDLR gene. This method is more accurate than the classical CRISPR-Cas9 technology and allows targeted point substitutions in the DNA sequence by hydrolytic deamination, avoiding double-strand breaks (Hu et al., 2018; Porto et al., 2020). This method has already been successfully used to generate isogenic cell lines to model a number of diseases, namely Alzheimer’s disease (APOE4 gene sequence correction), sickle cell anaemia (β-globin gene), Hutchinson–Gilford progeria (lamin A gene), hereditary haemochromatosis (HFE gene) and some cancers (TP53 gene) (Komor et al., 2016; Gaudelli et al., 2017; Koblan et al., 2021; Newby et al., 2021).

Verve Therapeutics is conducting the first clinical trial using base editing for FH therapy with the drug VERVE-101 starting from 2022 (ClinicalTrials.gov ID NCT05398029). PCSK9 is the target gene for VERVE-101. Base editing of the gene disrupts PCSK9 protein synthesis, which in turn disrupts LDLR receptor degradation and leads to a reduction in plasma LDL concentrations (Rothgangl et al., 2021). However, this drug is not effective in FH patients with pathogenic allelic variants in LDLR that disrupt receptor synthesis or release to the cell surface, such as c.530C>T and c.1054T>C. In this context, the development of new FH cell models using safer cell genome editing systems remains a priority.

The isogenic iPSC cell lines of an FH patient we have obtained can be used to study dysfunction of relevant differentiated derivatives, such as endotheliocytes and hepatocytes, involved in FH manifestation, as well as to develop approaches for screening pharmacological compounds that are potential drugs for effective FH therapy.

There are known examples of clinical trials for some drugs selected using iPSC-based cell models, namely amyotrophic lateral sclerosis, progressive ossifying fibrodysplasia, Pendred syndrome and Alzheimer’s disease (Okano, Morimoto, 2022). This approach appears to make economic sense as it allows first-line screening of compounds without the need for less relevant and more expensive animal models.

Despite the many advantages of using iPSCs for disease modelling, there are a number of limitations and challenges that need to be overcome in further research. A major drawback is that many of the differentiated derivatives obtained from iPSCs are functionally immature (Brooks et al., 2022). To address this problem, approaches are being developed to profile differentiated iPSC derivatives at the transcriptomic level at different stages and to identify robust criteria to assess the maturity of cell models (Subramanian et al., 2019; Kannan et al., 2021). Another challenge is to create reproducible, relevant models that integrate multiple iPSC-derived cell types and reflect disease pathogenesis under cell-cell interactions. Overcoming this involves using cellular organoids or assembloids (Brooks et al., 2022). However, this does not solve the problem of efficiency and reproducibility due to fluctuations caused by self-organisation within organoid systems. Bioprinting technology with a defined number of viable cells and their interaction pattern is currently being considered as a promising approach to improve the reproducibility of complex integrated cell models (Renner et al., 2020; Hofer, Lutolf, 2021; Lawlor et al., 2021).

We hope that using our cell models based on isogenic iPSC lines from patients with FH will help to better understand the mechanisms of disease progression and to develop effective drugs, increasing treatment efficacy and improving the quality and duration of patients’ lives.

## Conclusion

In the present study, we obtained and characterised in detail the genetically modified iPSC line ICGi036-A-1, which was derived from an isogenic line of an FH patient with compound heterozygosity for pathogenic and likely pathogenic allelic variants of the LDLR gene, namely c.530C>T (p.Ser177Leu) and c.1054T>C (p.Cys352Arg). The resulting line had a corrected c.530C>T position to the reference c.530C. The new iPSC line will be used to generate relevant differentiated derivatives to study FН manifestations and to develop FH- targeted therapeutic approaches.

## Conflict of interest

The authors declare no conflict of interest.
